# Vascular anomaly in bilateral ectopic kidney: a case report

**DOI:** 10.1186/1757-1626-3-5

**Published:** 2010-01-05

**Authors:** Gokhan Gokalp, Bahattin Hakyemez, Cuneyt Erdogan

**Affiliations:** 1 Department of Radiology, Uludag University Medical Faculty, Gorukle, Bursa, Turkey

## Abstract

Ectopic kidney occurs as a result of a halt in migration of kidneys to their normal locations during embryonal period. While kidneys ascend through pelvis, they receive new branches from vessels (iliac and aorta) close to them. When they reach the highest point, they receive new branches from aorta and the former branches degenerate. Renal vessels do not degenerate in the ectopic caudal kidney, more than one accessory and polar arteries may arise. In various studies, a possibility of association between presence of multiple renal arteries and hypertension, has been reported. We aimed to present a case with bilateral ectopic kidney and vascular anomaly associated with hypertension and renal dysfunction.

## Introduction

Ectopic kidney is described as abnormal localization of kidney due to a developmental anomaly and it occurs as a result of a halt in migration of kidneys to their normal locations during embryonal period. More than one anomaly can occur at the same time. While kidneys ascend through pelvis, they receive new branches from vessels (iliac and aorta) close to them. When they reach the highest point, they receive new branches from aorta and the former branches degenerate. If those vessels do not degenerate in the ectopic caudal kidney, more than one accessory and polar arteries may arise [1,2]. Hypertension is more frequently encountered in cases with more than one renal arteries[3-6]. In the present report, we aimed to present a case with bilateral ectopic kidney and vascular anomaly.

## Case Report

**Figure 1:**
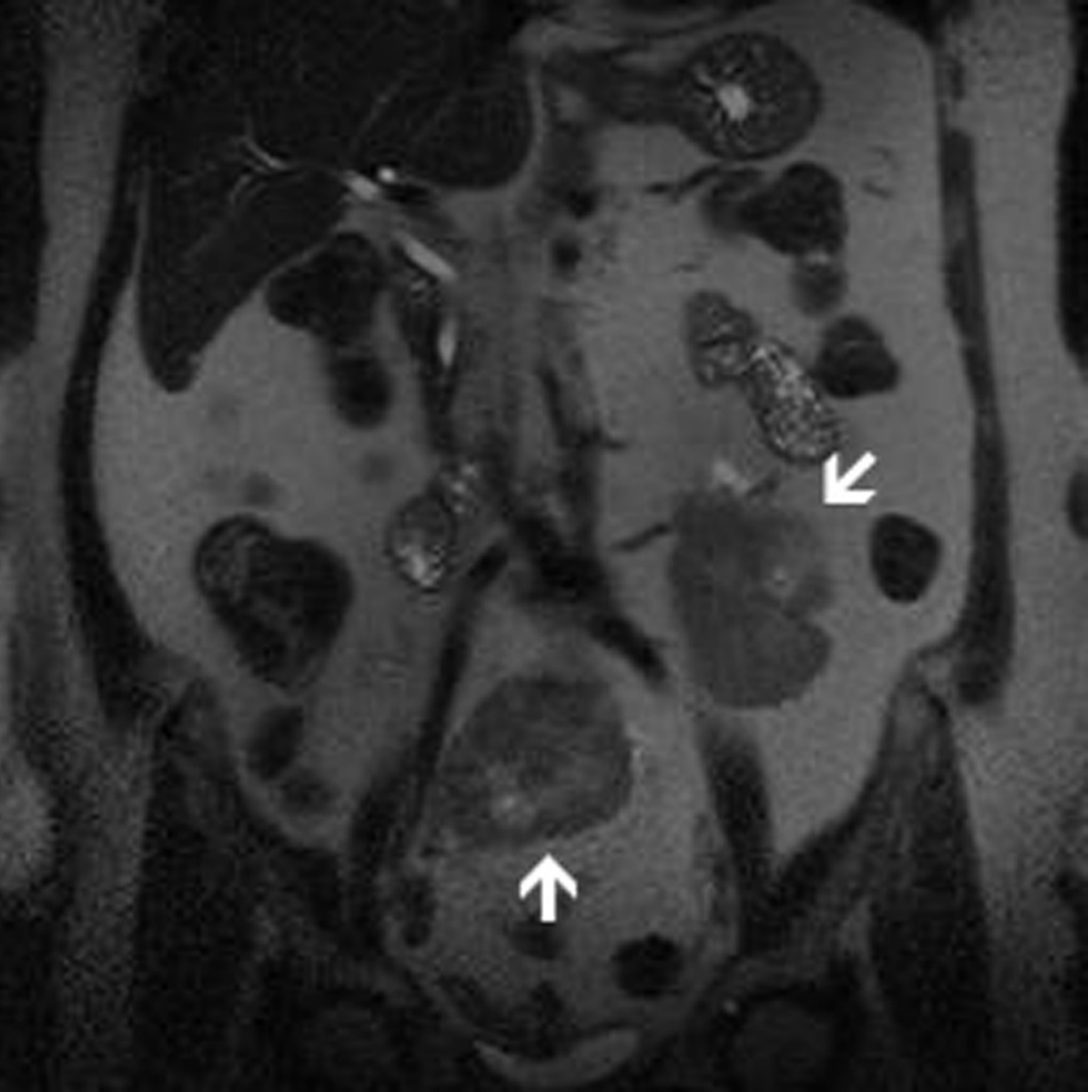
**T2-weighted half Fourier single-shot turbo spin echo (HASTE) sequence showing bilateral renal ectopy (arrows)**.

**Figure 2:**
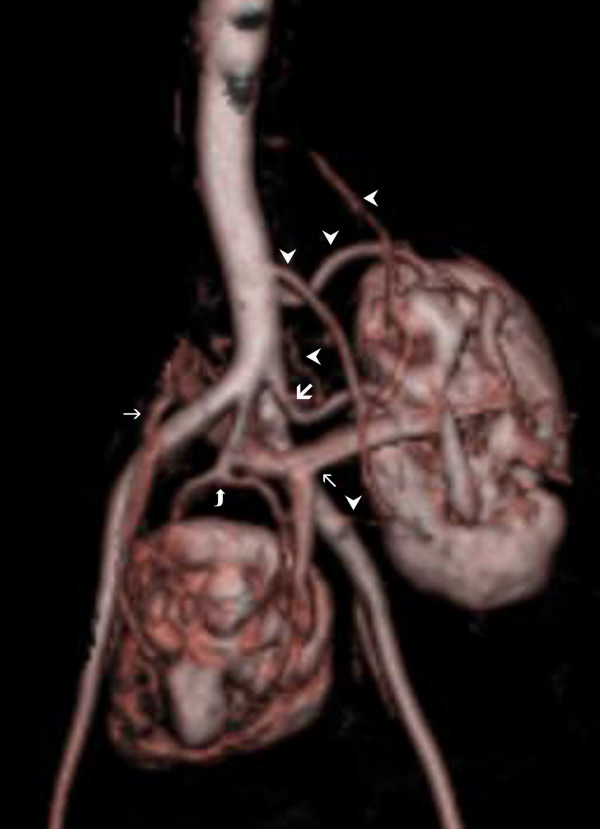
**MRI contrast angiography (3D FLASH) "volume rendering" image**. Accessory artery originating from iliac bifurcation of left kidney (thick arrow) and 5 polar (capsular) arteries can be seen (arrow heads). Most superior polar artery originating from distal SMA, courses through lower pole of left kidney. A renal artery branching from right common iliac artery starting point, divides into 2 branches and reaches renal pelvis (bended arrow). No polar artery is seen in this kidney. The kidney in the mid level has 2 renal veins, and the left vein is joining with the left renal vein and opening into vena cava interior (thin arrow).

The 67-year-old female patient who has been receiving medication for hypertension for 25 years presented with dysuria. Blood pressure was 160/90 mmHg. The patient was not investigated for reasons of hypertension before. Medical history and family history showed no remarkable findings and laboratory tests were carried out. Blood urea and creatinine levels were 120 mg/dL, 2.2 mg/dl respectively. GFR was detected before contrast media application and it was normal. Because there was no evidence of proteinuria in urinary analysis of the patient, magnetic resonance imaging (MRI) angiography was applied for renovascular pathology. The imaging protocol for evaluation of the kidney at our institution on a 1.5-Tesla MRI system (Magneton Vision, Siemens Medical Solutions) consists of the following sequences: 1. T2-weighted half Fourier single-shot turbo spin echo (HASTE) sequence (TR/TE, 4.4/90; flip angle, 150°; matrix size, 256 × 160; slice thickness, 5 mm) coronal. 2. T1-weighted three-dimensional gradient echo sequence (FLASH) for dynamic (TR/TE, 4/1.6; flip angle, 30°; matrix size, 256 × 150; slice thickness, 3 mm) coronal, using intravenous macrocyllic contrast agent [Dotarem (meglumin gadoterat), 20 ml)], immediately followed by two breath-hold periods with two scan series per breath-hold. In this way pre-contrast and postcontrast images in arterial and venous phase are obtained. An MRI contrast angiography revealed 2 different ectopic kidneys with hila facing ventral aspect, one of which was in the middle of two iliac arteries in the pelvic region and the other localized at a higher spot adjacent to left main iliac artery and aorta (Figure 1). Left kidney had an accessory artery originating from the proximal of the left main iliac artery of left kidney, and 5 polar (capsular) arteries. The mid level kidney had a renal artery which was originating from the iliac artery with findings of early branching. Mid level kidney had 2 renal arteries and left vein was joining with left renal artery and opening to vena cava inferior at a low level (Figure 2). The proper medical treatment was planned and the case was taken under monitoring. The patient was prescribed antihypertensive drugs and was later followed with renal function tests.

## Discussion

Urinary tract, originating from mesoblast and expanding from cranial to caudal, completes its development after 3 developmental phases: pronephros, mesonephros, and metanephros. The third phase gives rise to the permanent kidneys. Initially, they localize at ventral portion of sacrum and pelvis, close to each other. Along with abdominal and pelvic growth and decrease of body inclination, they follow an ascending route and reach their permanent sites by coming in contact with the adrenal gland at 9th week. First, hila face ventral aspect, however, during ascent, it displays 90 degree medial turn. The hilus of a kidney that attained its final permanent position, faces anterolateral aspect. During ascent, they proceed through a bifurcation formed by umbilical arteries. If one of the kidneys fail to pass this point, it remains within the pelvis next to the common iliac artery. This kidney is called as pelvic or ectopic kidney, and is associated with malrotation [1,2]. Renal ectopy is a relatively common congenital anomaly and can not be noticed unless it causes any symptomatology. It is most commonly presented with unilateral, pelvic localization along with a minimal left side and male predisposition [7].

At mesonephros, lateral intersegmental arteries, which are called as urogenital rete arteriosum, provide vascularization. Those arteries build a vascular net that supports adrenal glands, kidneys, and gonads, at both sides of the aorta between 6th cervical and 3th lumbar vertebrae. In other words, kidneys receive branches from blood levels close to them during their ascent. First, renal arteries receive branches from common iliac artery. As the ascent proceeds, kidneys start to receive branches from distal end of the aorta. When they reach the highest level, they receive new branches from aorta, and under normal conditions, the former vessels degenerate and eventually disappear. Permanent renal artery develops from one persistent branch of urogenital rete arteriosum [1].

The differing origins of renal arteries and commonly encountered variations, are explained by development of mesonephric arteries. Insufficient degeneration of mesonephric arteries, leads to presence of more than one renal artery [1,5,6]. Renal artery variations are categorized in 2 groups: "early branching" and "extra renal arteries". While main renal arteries divide into segmental branches at hilus level, a branching occuring more proximal to hilus, is called "early branching". Extra renal arteries are grouped in 2 as follows: hilar (accessory) and polar (aberrant) arteries. While hilar arteries enter kidney through hilus with main renal artery, polar arteries penetrate kidney directly through the capsule from outside of the hilus [8]. In the present case, an accessory artery of the left kidney, originating from starting point of left common iliac artery, and 5 polar (capsular) arteries, were present. The kidney localized at mid level, had a renal artery originating from right common iliac artery, and early branching was present.

Extra renal artery prevalence differs depending on social and ethnic characteristics. Its prevalence is reported to be between 9% and 76% (mean value: 28%) [8,9].

Multiple renal veins constitute the most common venous variant and are seen in 15-30% of individuals. Occasionally, single renal vein may divide into branches prior to joining inferior vena cava [10]. In the present case, the kidney localized in the mid level of pelvis had 2 renal veins and the left vein was joining with left renal vein and eventually opening into vena cava inferior.

Renovascular hypertension is defined as a syndrome of arterial hypertension induced by renal perfusion pressure secondary to a vascular lesion [11]. In various studies, a possibility of association between presence of multiple renal arteries and hypertension, has been reported [3,6]. A study conducted by Glondy B et al. [5], showed high plasma renin activity in cases with multiple renal arteries, and the authors highlighted that this may lead to a predisposition for hypertension. According to a hypothesis, accesory renal arteries usually have a longer length and a smaller diameter compared to the main artery, and the renal segment supported by this artery display lower blood pressure which cause hypertension by stimulating renin secretion. However, the study performed by Gupta A et al. [12], showed no association between hypertension risk and accessory renal arteries. In the present case, the association of multiple renal arteries and hypertension/renal failure, is not clear and open to discussion. However, due to presence of studies reflecting an association between presence of multiple renal arteries and hypertension, we believe this possibility should be considered, as well.

CT and MRI are comparable for evaluating renal disease in the majority of patients. MRI angiography was performed in the present case becuse of renal disfunction. There are circumstances in which MRI is, however, the procedure of choice, which includes, patients with allergy to iodine contrast, complicated, particularly calcified, renal cystic lesions and renal masses in which CT images are difficult to interpret. Furthermore, MRI can be used as a problem-solving modality when the CT findings are nondiagnostic. Attempts are being made to use MRI for imaging of renal function, including perfusion, glomerular filtration rate and intrarenal oxygen measurement [13].

In conclusion, bilateral renal ectopy associated with malrotation is an uncommon anomaly. Due to failure of normal renal ascent and regression, accessory and polar arteries may occur and venous anomalies may accompany. Those arterial anomalies may cause renovascular hypertension and even lead to renal insufficiency. However, further detailed studies are required about this relationship.

## Consent

Written informed consent was obtained from the parents of the patient for publication of this case report and accompanying images. A copy of the written consent is available for review by the Editor-in-Chief of this journal.

## Competing interests

The authors declare that they have no competing interests.

## Authors' contributions

GG collected the patient history data, edited the text and wrote the paper. BH chose the radiology images and provided descriptions of same. CE was the overall supervisor and assisted with editing the manuscript. All authors approved and read the final manuscript.
